# Supplementary material for: Every Step Counts—How Can We Accurately Count Steps with Wearable Sensors During Activities of Daily Living in Individuals with Neurological Conditions?
Source: Sensors (Basel). 2025 Sep 11;25(18):5657. doi: 10.3390/s25185657 (PMC12473868; doi:10.3390/s25185657)
Supplement: Supplementary file 1 [file sensors-25-05657-s001.zip › sensors-3803573-supplementary.pdf]

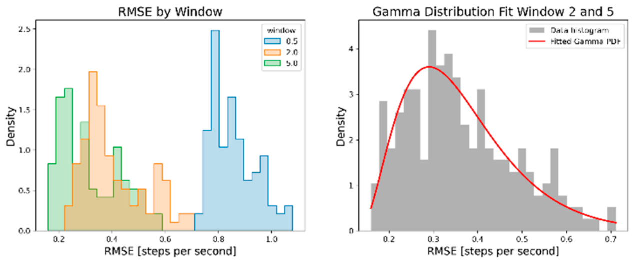

Figure S1. Histogram of RMSE for window length. Left side: Histogram of the Root Mean Square Error (RMSE) for the three different window lengths 0.5 second (blue), 2 seconds (orange), and 5 seconds (green). The RMSE for the 0.5 second window is substantially larger than for the other two window length, creating a bimodal distribution. Right side: Histogram of the RMSE for the two remaining window lengths (2 and 5 seconds, after excluding 0.5 second window) including the fitted gamma probability-density function (pdf) of the model.

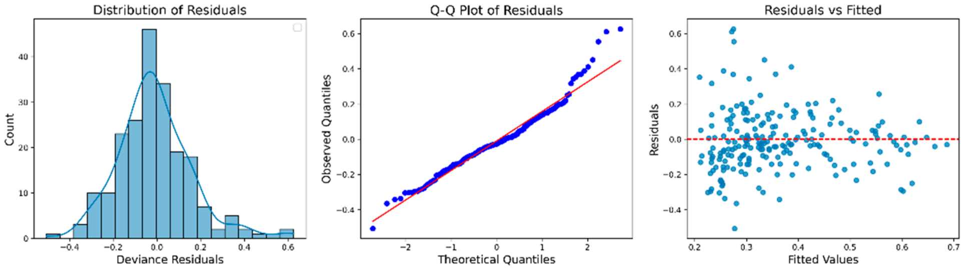

Figure S2. Visualizations of generalized linear model fit. Distribution of model residuals (left), quantile-quantile plot of the observed versus theoretical residuals (middle) and residuals against actually fitted values (right) to assess the fit of the generalized linear model to analyse the optimal sensing and analysis configuration (primary aim).

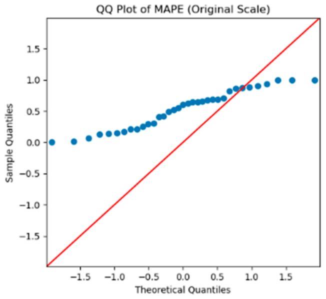

Figure S3. Assessing normal distribution for algorithm comparison. Quantile–quantile (Q–Q) plot of the Mean Absolute Percentage Error (MAPE) comparing the sample distribution of the algorithm comparison (secondary aim) against the theoretical normal distribution. Deviations from the diagonal line indicate departures from normality.

Table S1. Detailed study protocol with all tasks.

#	Task description	#	Task description	#	Task description
1	Rest in bed	14	Fetch the keys	27	Get bread and fruits
2	Put on shoes and glasses	15	Switch off the light	28	Get utensils (spoon, milk)
3	Put on a shirt	16	Open the door	29	Transport tray to table
4	Walk to table	17	Leave the room	30	Set table
5	Write some notes by hand	18	Close the door	31	Snack time
6	Open a closed bottle	19	Walk to the stairs	32	Stack utensils on tray
7	Walk to bathroom	20	Walk up the stairs (elevator)	33	Transport tray back
8	Turn the light on	21	Walk to the kitchen	34	Tidy up
9	Brush teeth and comb hair	22	Hang on jacket	35	Go back to table
10	Wash and dry hands	23	Rest on chair	36	Swipe the table
11	Sit on couch, turn TV on	24	Pour water and drink	37	Go to the sofa area
12	Walk to closet	25	Walk to kitchen	38	Sit down
13	Put on a jacket	26	Prepare tea/coffee	39	Walk back to bedroom

Table S2. Feature list

#	Feature description	#	Feature description	#	Feature description
1	Mean of vertical axis	7	RMS of vertical axis	13	Magnitude of dominant frequency of vertical axis
2	Mean of anteroposterior axis	8	Jerk of vertical axis	14	First positive autocorrelation peak of vertical axis
3	Standard deviation of vertical axis	9	Correlation between vertical and anteroposterior axis	15	Time at which autocorrelation of vertical axis reaches maximum
4	Standard deviation of anteroposterior axis	10	Correlation between vertical and mediolateral axis	16	Number of zero crossings of autocorrelation of vertical axis
5	Energy of vertical axis	11	Correlation between mediolateral and anteroposterior axis	17	Number of peaks in vertical axis signal
6	Energy of anteroposterior axis	12	Dominant frequency of vertical axis	18	Number of peaks in vertical axis magnitude
